# Proposal for a Protocol and a Handmade Arduino-Based and Open Source Device for Measuring the Residual Charge of Alkaline Batteries in View of an Attempt to Recharge Them

**DOI:** 10.3390/mps9020066

**Published:** 2026-04-19

**Authors:** Giovanni Visco, Maria Pia Sammartino, Angela Marchetti, Mauro Castrucci, Mauro Tomassetti

**Affiliations:** Department of Chemistry, University of Rome, “La Sapienza”, P.le A. Moro 5, 00185 Rome, Italy; giovanni.visco@fondazione.uniroma1.it (G.V.); angela.marchetti@uniroma1.it (A.M.); mauro.castrucci@libero.it (M.C.); mauro.tomassetti@uniroma1.it (M.T.)

**Keywords:** Arduino project, measure of residue charge, alkaline battery, electrochemistry

## Abstract

Portable devices are powered in direct current (DC) or by batteries (primary battery), accumulators (secondary battery), and now supercapacitors, which can also be used for energy storage. The European Portable Battery Association states that approximately 239,000 tons of batteries were placed on the market in the European Economic Area (EEA) plus Switzerland in 2022. Even if they were all disposed of correctly respecting the 3R paradigm (Reduce, Reuse and Recycle), non-rechargeable batteries create an environmental problem because they do not discharge completely with an obvious waste of energy. Secondary batteries and supercapacitors can be recharged because they use reversible chemical/physical processes while primary batteries cannot be recharged because they are based on irreversible redox reactions; nevertheless, it is possible to try to recover their residual charge if this is higher than a threshold beyond which the reactions can be reversible. The most used batteries are alkaline zinc/manganese dioxide and they are non-rechargeable; an inappropriate recharge attempt can lead to serious harm to the operator and the environment. This paper describes a simple Arduino-based circuit and the protocol to measure and graph the residual charge of an alkaline battery in order to establish if it can be recharged. The circuit, design, the Arduino Uno R3 sketch (i.e., microprocessor software) and the full protocol are here presented under the open source license (Copyright Creative Commons Public license, CC BY-NC-ND 4.0 EN) so that they could become a pilot system and then a commercial product. The residual charge of 158 batteries, obtained after discharging those that, by eye, appeared damaged, was measured. Results evidenced that 49% of batteries had a residual voltage, under low load, between 1.2 and 1.6 V, making them good candidates for a recharge attempt.

## 1. Introduction

**Figure 1:**
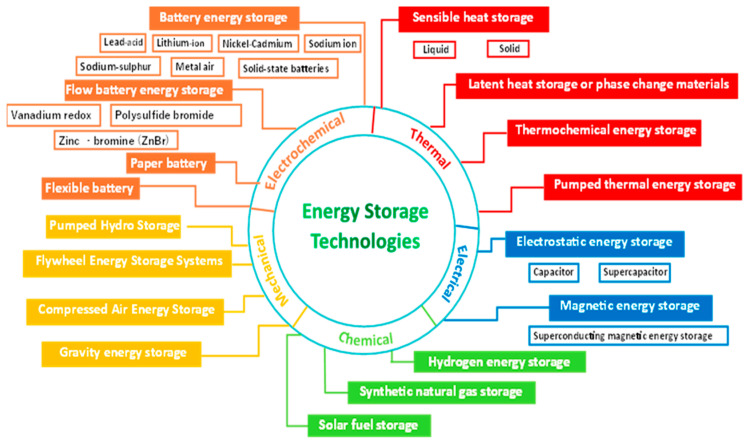
Current state of technologies for energy storage copied from a paper of Ioana-Cristina Badea et al.; the original paper is under CC-BY license and the complete reference is in [3].

Portable devices are often powered by batteries (primary or secondary battery), and now supercapacitors, which can also be used for energy storage [1]. Approximately 239,000 tons of batteries were placed on the European market in 2022 [2]. Especially for portable instruments that require electricity to operate, the alternative to connecting to the mains is a battery of the appropriate voltage. Batteries can be divided into two categories: non-rechargeable, called primary battery, and rechargeable, called secondary battery or also storage battery. Other classifications are possible; their description is beyond the scope of this article and in any case is not possible given the continuous invention of new typologies or the redesign of existing models. Nevertheless, we can cite the exhaustive classification by Ioana-Cristina Badea et al. [3] (Figure 1) for the current state of the art. The aim of our research is instead an evaluation of the quantity of residual charge, called residual energy, due to the incomplete discharge of non-rechargeable batteries. If in secondary batteries this residual charge is necessary for the correct functioning of the battery and to preserve its efficiency, in a primary battery it is lost with evident economic and, above all, environmental damage [4]. The value of the residual charge depends on the energy required by the instrument using the battery, so, for example, the one used in a toy stops working with a sufficiently high residual charge and, maybe, it can be used in a device that requires less energy or can be recharged. So, our aim is to propose a protocol using a handmade economic instrument, Arduino-based, presented under the open source license (Copyright Creative Commons Public license, CC BY-NC-ND 4.0 EN [5]), for a safe measure of the residual charge with the main aim of determining whether the battery can be recharged (running research). As a fact, even if they are called “not rechargeable batteries”, the chemical reactions occurring inside an alkaline battery are reversible if the residual charge is higher than 60% [6] and some studies estimate that discarded single-use batteries can contain up to 50% residual energy [7]. Other researchers measured the energy loss due to the under use of batteries to evaluate the corresponding environmental and economic impact on a national scale; Sabbaghi et al. [8] found that discarded AA/LR6 alkaline batteries retain about 13% of their initial energy; even though it is small, this residual energy should not be underestimated given the amount of portable batteries on the market. A study by the European Portable Battery Association indicates that in 2018 approximately 239,000 tons (or 11 billion units) of portable batteries were placed on the market in Europe plus Switzerland [9] with an increase of +0.8% compared to 2017; this increase was independent of the population increase which was +0.3% and corresponds to approximately 22 portable batteries per person compared to 19 in 2013 [9]. In 2018 it was also reported that over 110,000 tons of portable battery waste were collected (+6.2% compared to 2017) corresponding to a collection rate of 47.4%, compared to 45.5% in 2017 and 46.3% in 2016. It must also be considered that when, as in most cases, a pack of multiple batteries is needed, it may happen that only one is actually flat but all are wasted. Such a large quantity of products, of various types, requires further Life Cycle Assessment (LCA) studies to evaluate the impacts arising from all phases of the life cycle of the manufactured batteries [10]: extraction/production of the constituent materials, transport, recharging when possible, disposal, and recovery of the materials themselves. These studies show that during the production phase, the environmental impact of primary batteries is higher than that of storage batteries but in the life cycle the environmental impact of secondary batteries increases and becomes very high if the number of recharges is too low (<20 recharge cycles) before the end of life. Even with 150 recharges, NiMH batteries are not at all better from an environmental point of view than alkaline batteries and, anyway, the use of rechargeable batteries for at least 50 cycles is recommended to decrease their environmental impact [11] and technological attempts to improve them are an object of research [12]. During the discharge of a battery the concentrations of the species involved in the electrochemical reactions on both electrodes vary with the discharge time, with the current required, and with the intermittency of the request. The chemical reactions inside a common battery show a recovery effect that allows a further supply of energy even if for a very short period, for example a remote control that stops working and starts working again the next day even if only for few channel changes.

Various methods have been developed to measure the residual energy of any battery including impedance spectroscopy in alternating current at various frequencies [13] depending on the type of battery. Some papers compare state of charge (SoC) measurement using electrochemical impedance spectroscopy with other methods similar to the one we here propose, i.e., voltage measure at constant load [14]; for example, the comparison of Figures 3, 4, and 12 of a paper by Diard [14] with Figures 1 and 6 of a paper by Franke-Lang [15] shows similar results using the two techniques. Any instrument for the measurement of residual energy requires the use of a microprocessor or rather a microcontroller (similar to those in Arduino (Arduino llc., Ivrea, Italy), Raspberry Pi (Raspberry Pi Holdings plc, Pencoed, UK), BeagleBone (GHI Electronics, Madison Heights, MI, USA), ESP32 (Espressif Systems Co., Ltd., Shanghai, China), Teensy (SparkFun Electronics, Boulder, CO, USA) that not only runs specially written software but also manages input–output, measures voltages and drives external actuators.

**Figure 2:**
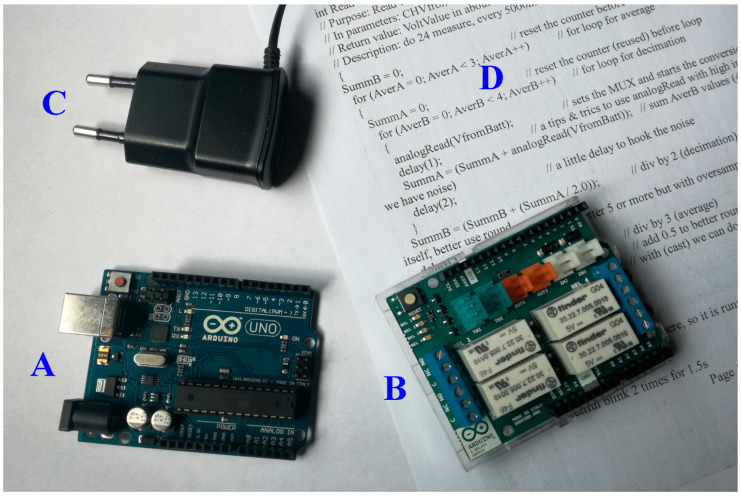
Components needed for an electrochemical measurement, (**A**) a microcontroller, (**B**) an interface/converter/actuator, (**C**) a power supply, (**D**) the management software.

One of the strong points of the Arduino boards (A in Figure 2) is the possibility to load the management software (D in Figure 2, called Sketch) inside the microcontroller, Atmel AVR ATmega32P [16], over and over again until the right implementation is found, and to be able to operate, measure and display (also with the help of a special interface called Shield, B in Figure 2) the results without being connected to the computer or even connecting to external software, guaranteeing complete independence, portability and good precision of the times and values acquired [17,18].

Arduino can be an ideal choice for measuring the residual energy of any battery, either alone or with some external circuits, as in our case, or together with other software, e.g., LabView [19], controlling both the discharge and/or charge voltage and current. When charging, the use of a microcontroller is almost essential to keep the battery within the Safe Operation Area (SOA) [19].

Given the importance of managing the SOA of a battery, the major integrated circuit manufacturers have over time developed circuits [20] to control it, which, regardless of the battery chemistry, alkaline, nickel–cadmium (NiCd) or nickel–metal hydride (NiMH), measure for the single cell both the discharge voltage, which can be as low as 0.8 V–0.9 V, and where applicable the charge voltage between 1.35 and 1.65 V.

This work describes a simple and open source protocol for measuring the residual charge of a common alkaline battery using as microcontroller board Arduino UNO R3 with some very simple external circuits. It constitutes a valid alternative to more complex and expensive methods such as Nyquist plot, electrochemical impedance spectroscopy (EIS) and distribution of relaxation times (DRT) [21].

## 2. Experimental Design

### 2.1. Methods

First of all, it is necessary to take into account the extensive legislation regarding the construction and marketing of all types of batteries, to which over time “environmental” legislation has been added as they contain metals and chemical substances that are dangerous for the environment and for users. It is also necessary to mention the IEC (International Electrotechnical Commission) 60086-x series that standardizes primary battery measurements as follows: Part 1, dimensions, nomenclature, terminal configurations, markings, test methods, typical performance [22]; Part 2, physical and electrical specifications, discharge test conditions [23]; Part 3, specifications for watch batteries [24]; Part 4, specifies tests and requirements for primary lithium batteries and safe operation [25]; Part 5, specifies tests and requirements for primary batteries with aqueous electrolytes [26]; Part 6, testing the environmental performance of batteries [27].

Before studying batteries, we recommend reading a number of other norms, such as: ANSI C18.xx, UL 1642-2054, IEEE 1625-1642, USNEC 480, GB/T18287-289.

**Figure 3:**
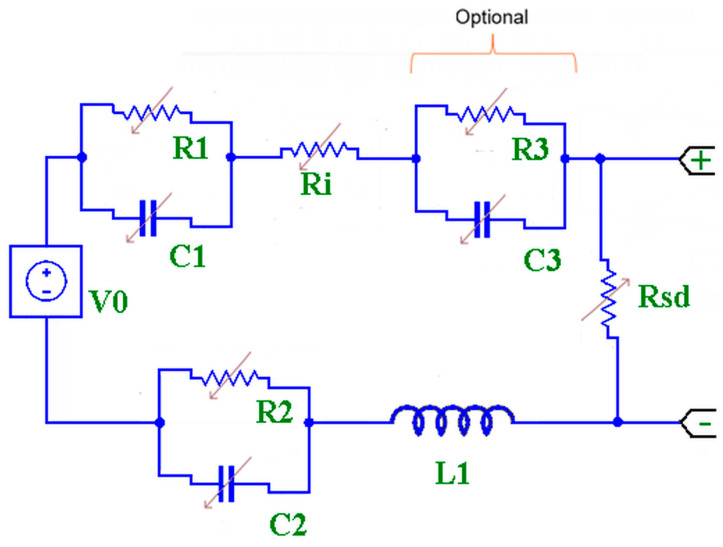
Simplified equivalent circuit of a battery. V0: theoretical battery voltage, R1-C1 and R2-C2: parasitic resistor and capacitance on positive and negative electrode respectively, R3-C3: parasitic component from “junctions”, Rsd: self-discharge resistor, Ri: internal resistor series with V0, L1: internal inductance.

To perform a measurement that truly describes the state of charge of the battery we need to know its internal circuit elements. Figure 3 draws the equivalent simplified circuit of a battery redrawn from the references MatWorks and BioLogic [28,29].

Values of each component, obviously not real, vary with temperature, state of charge and the request for continuous or pulsed current.

The norms cite at least four cases for the discharge of a battery, at constant load resistance, at constant current, at constant power and the most complex case of short current requests over time (pulsed discharge).

**Figure 4:**
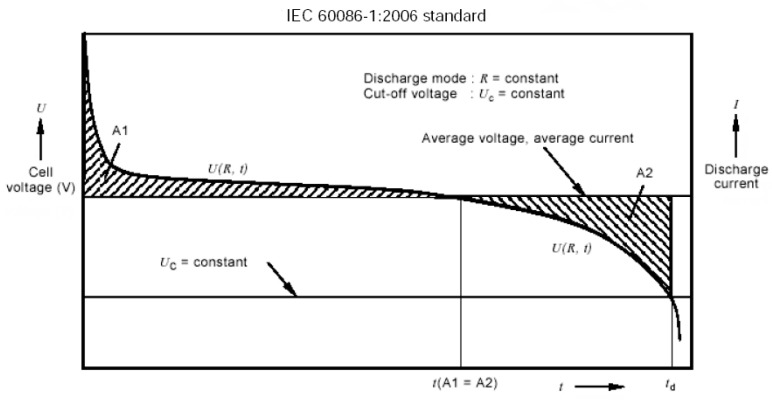
Schematic standard discharge voltage of an alkaline battery under the application of a constant load (R) (Figure 2 in IEC 60086-1 [22]).

To measure the SoC we chose the constant-resistance load method because it is simple and often cited in the literature [29,30]. We extracted Figure 4, which shows the typical discharge phase of a primary battery as a function of time with constant R load from the IEC 60086-1 [22]. It shows a rapid potential drop on the load application, a nearly constant potential until the end of phase A1, and a more rapid drop in phase A2 until reaching the cut-off normally set for alkaline batteries at 0.9 V. Some devices, such as, for example, a wall clock or a TV remote control, do not respect the cut-off and stop working when the battery is almost completely discharged.

If the cut-off is met, a significant residual charge remains inside the battery; we can cite some works that attempt to recover it [7,31], using Arduino’s ATmega328p as a microcontroller.

**Figure 5:**
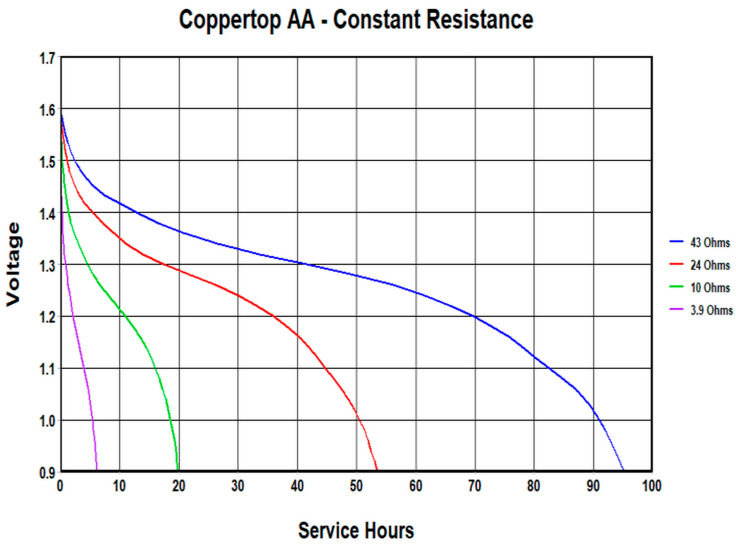
Typical discharge rate at constant R of an alkaline battery; data extracted from AA Duracell Ultra battery datasheet, cited from [33] with permission.

Based on Figure 4 and the relative mentioned norm, the choice of load resistor as well as the measurement time are crucial factors for proper residual charge diagnosis. Manufacturers’ datasheets for AA/LR6 batteries [32,33] are an aid for these choices; just as an example, Figure 5 shows the voltage obtainable over time as a function of the constant load resistance [30].

**Figure 6:**
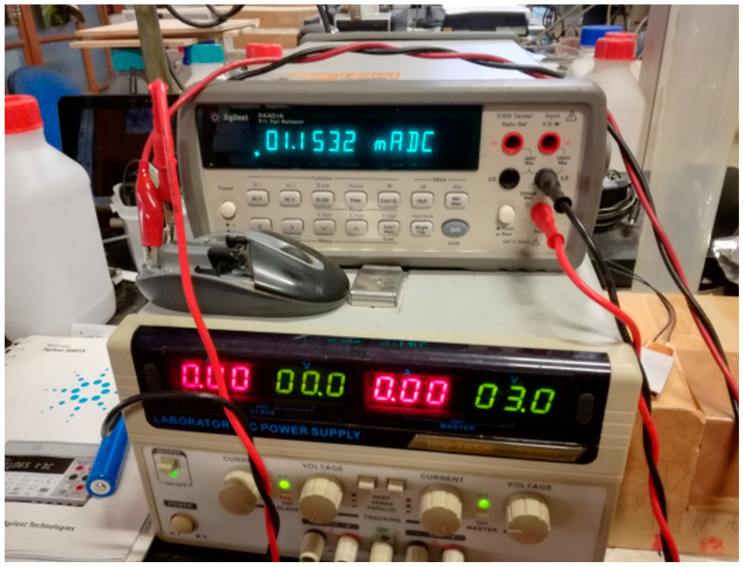
Measure on a top brand wireless mouse, static, with 3 V of power supply and 1.15 mA of current (2601 Ω calculated).

**Figure 7:**
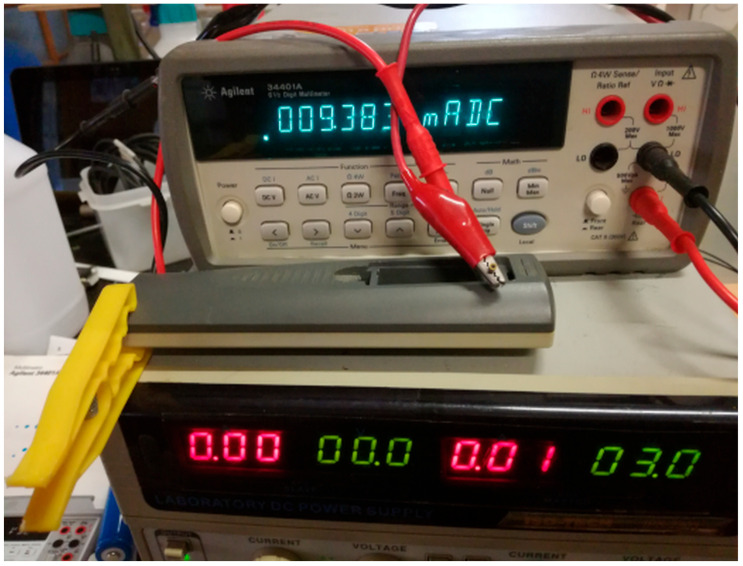
Measure on a TV remote control supplied with 3 Vdc using a current of 9.3 mA when sending data (320 Ω calculated). The yellow clamp presses the power-off button continuously.

Figure 5 shows that, obviously, the discharge time increases as the Rload increases; so, in order to discharge the battery under measurement (Device Under Test, DUT) as little as possible and at the same time to have a voltage curve in few seconds, we had to optimize the R load. At this aim a series of measurements was carried out in the laboratory on small low-power devices. As an example, Figure 6 and Figure 7 show those relating respectively to a wireless mouse of a well-known brand and to a TV remote control; a laboratory power supply set to 3 Vdc and a quality voltmeter were used. The resistances calculated for the two devices, respectively 2601 and 320 ohm, were found to be too high and would produce a too low current decay; therefore, also considering the discharge curves in Figure 5, we used the same apparatus and the sketch (see next Figure 11) to follow the discharge of six batteries (randomly choose between those sampled but discharged after these tests) with a series of resistances in the 50–200 ohm range. For each battery the following procedure was performed: measure under a 200 ohm Rload, after 24 h measure under a 100 ohm Rload, after 24 h measure under a 50 ohm Rload; results are reported in the file *Alkaline-measure-50-100-200.xls* of the Supplementary Materials and suggested to us to use a 100 ohm Rload.

The apparatus in Figure 6 and Figure 7 was also used for a bits vs. volts calibration (in triplicate) of our handmade instruments. To do this, we varied the supply voltage applied to the battery holder and read the bit values produced by Arduino and the sketch. We used Arduino UNO R3, Atmel MEGA328p SMD, and 11-bit oversampling to read the bits and different laboratory benchtop instruments to apply and read the voltage; specifically, we used two different DC power supplies (HP E3611A, TTi EX354D and three voltmeters (Agilent 34401A, HP 34401A, Philips PM2521) (respectively Hewlett-Packard Company, Loveland, CO, USA; Thurlby Thandar Instruments Ltd., Huntingdon, Cambridgeshire, UK; Agilent Technologies, Loveland, CO, USA; Philips Industrial & Electro-acoustic Systems Division, Eindhoven, The Netherlands).

The figures reported in the file *Alkaline-measure-3.3Vcalibration.xls* in the Supplementary Materials show that the three curves almost overlap, indicating good reproducibility of the Arduino-based instrument. Such data cannot be obtained for measurements on our real samples neither by successive measurements on the same battery, due to the, even if slight, decrease in voltage after each measurement, nor by measurements on different batteries precisely because they are different due to their different uses (obviously unknown).

The file also contains an educational description of the calibration procedure.

**Figure 8:**
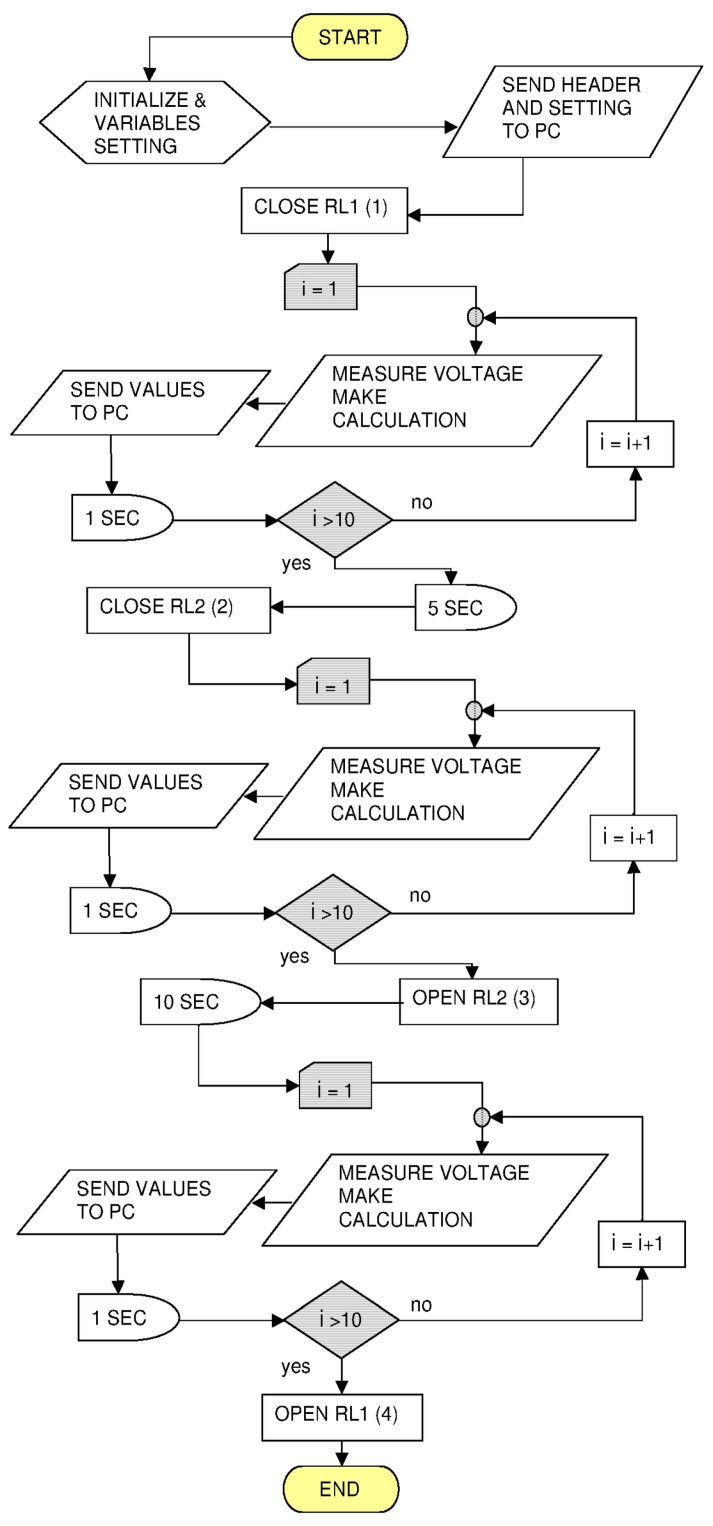
Flowchart of the Arduino sketch used to measure the state of charge of alkaline batteries.

**Figure 9:**
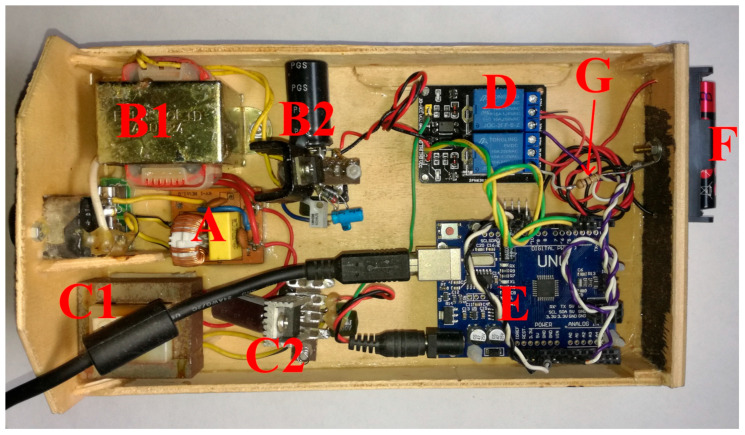
Handmade apparatus for measuring the residual charge under low load of an AA alkaline battery.

The flowchart in Figure 8 illustrates the measurement protocol to be followed using our handmade instrument (see next Figure 9) to obtain the discharge curves of the batteries, a more detailed text steps is presented in the file *flowchart-by-text.pdf* of the Supplemental Material.

**Figure 10:**
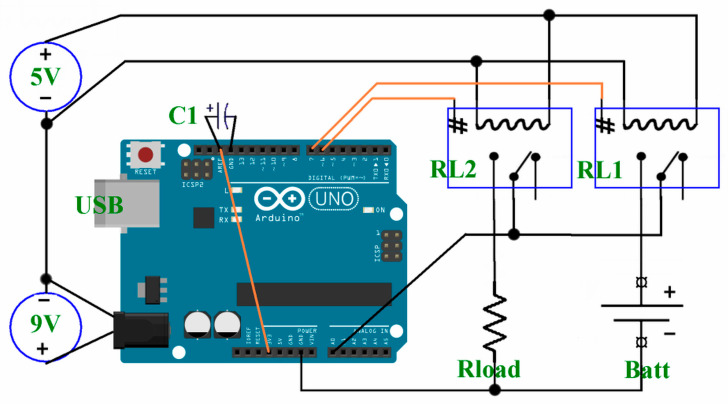
Scheme of the connections to Arduino of all the other electric components corresponding to the photo in Figure 9.

Referring to the circuit (see next Figure 10) in step (1) of the above flowchart the DUT is connected to the Arduino (with Zi > 100 MΩ, p. 319 of [34]). In step (2), the DUT is instead connected to the 100 ohm Rload and the discharge begins. In step (3), the Rload is disconnected and, after a delay, the V measurement continues. Finally, in step (4) one of the relays is opened to disconnect the battery. The “serial monitor” of the Arduino IDE collects the values sent to the PC. The text inside the window must be copied and pasted into a file saved with the name of the battery under measurement (we write a code with a permanent marker on each battery before the measurement); as an example, *Test-Battery-v1.1_AA-Panasonic-Everyday-01-29-G.txt* is reported in the Supplementary Materials.

Data of each battery are reported in the Supplementary Materials as an Excel file (*Alkaline-measure-extract-v1.1.xls*), containing tables and graphs produced by the single .txt files, which allows extracting the three phases of the measurement.

### 2.2. Materials

In our research we tried to respect the 3R paradigm, Reduce, Reuse, Recycle [35,36]; in fact, we looked for batteries still in a good state of charge and therefore suitable for a recharging attempt (our next research). Furthermore, where possible, for the assembly of the measuring instruments, we used parts, pieces, and electrical and electronic components recovered from old broken and non-repairable instruments or whose repair is economically unviable. Our measuring instrument is based on the one in Figure 2 but the relays in the shield (B in Figure 2), which could cost more than Arduino R3, are replaced by a pair of photocoupled relays for Arduino (see D in Figure 9).

Figure 9 shows the handmade apparatus built inside a recycled wooden box. In A we can see the connection to the 230 V mains and the ElectroMagnetic Interference filter, in B1 and B2 the 5 V stabilized power supply for the relays, in C1 and C2 the 9 V stabilized power supply for Arduino, in D the pair of relays, in E Arduino UNO R3, and in F a battery holder for AA and in G the 100 ohm 2 W resistor.

As an alternative to build the instrument with recycled parts, the main components are available at low cost; an example from an online Italian store (accessed in December 2025) is as follows:

D = https://www.robotstore.it/Modulo-rel%C3%A8-2-canali-DC-5V-per-Arduino-e-Raspberry-Pi accessed on 6 March 2026 (4.50 €).

E = https://www.robotstore.it/Arduino-UNO-R3-con-microcontrollore-ATmega328 accessed on 6 March 2026 (27.90 €).

C = https://www.robotstore.it/Alimentatore-da-parete-9V-2A-con-jack-per-Arduino accessed on 6 March 2026 (3.90 €).

B = https://www.robotstore.it/Alimentatore-da-parete-USB-5V-2-5A accessed on 6 March 2026 (4.90 €).

Figure 10 shows the simple circuit of the instrument part that in Figure 9 connects Arduino with the two relays and the DUT.

Theoretically, a single 9 Vdc power supply would be enough to run the entire system, since Arduino autonomously produces a stabilized voltage of 5 Vdc, required by the relays; unfortunately, the activation coil of a relay (black spiral in RL1/RL2) often requires a current greater than Arduino can generate. Furthermore, a double power supply allows using, by recycling them, relay modules already present in a research laboratory, perhaps 12 or 24 V. In Figure 10 we can see on the bottom left the power supply for Arduino (C1 and C2, Figure 9) and in the upper left the supply for the relays (B1 and B2, Figure 9), on the right part of the bottom the battery holder and the battery itself (F, Figure 9) and the load Rload (G, Figure 9), on the top the two relays RL1 and RL2 (D, Figure 9) and in the center Arduino with its connections. The connection between the 3.3 V pin and Aref, as well as C1, will be described in the software. To avoid, or at least limit, ground loops that could cause self-oscillations and/or unwanted noise, the battery, the load Rload, Arduino and the power supplies are connected to a common ground. The load Rload and the battery are connected to the Normally Open (NO) contact of the relays, so that, in the absence of power and without a sketch for the battery, the circuit is open. Both centers of the relays are connected to the Arduino measurement pin, A0; the sketch will move the contact from Normally Closed (NC), at rest, towards the battery or the load Rload.

Pins 6 and 7 of Arduino are connected to a relay input circuit that decouples this input from the external power supply and contacts to be able to use (respecting all safety requirements) the relays also for motors, 230 V power supply, etc.

### 2.3. Software

**Figure 11:**
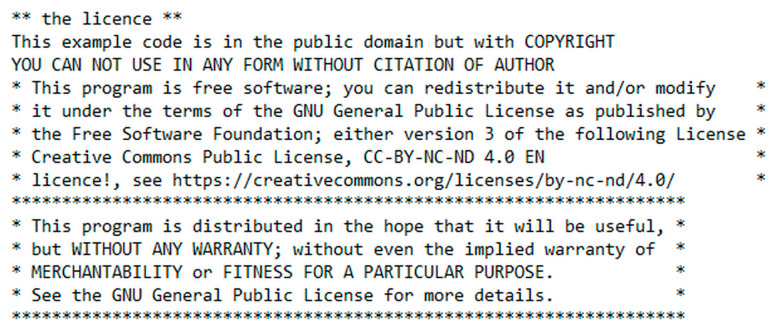
Our software, the sketch, the circuit and the instrument design are under copyright with the license CC-BY-NC-ND 4.0 [37]; here is a screenshot of part of the sketch code.

The software, the electrical circuit, the description of the operation and the information necessary for the realization of the instrument are described in this work and covered by copyright according to the open source license CC-BY-NC-ND 4.0 EN [37]; as a reference to this we show in Figure 11 some lines of the sketch.

The Atmel microcontroller should be programmed in MacroAssembler using the instruction set provided in the microcontroller datasheet [34]. Assembler has a large set of instructions and a logic related to speed of execution and operation rather than “simplicity”, which is usually not advisable except in special cases. A first level of simplification for the programmer is the use of a compiler that translates the instructions from a programming language (Fortran, Lisp, Ruby, Rust, etc.) into assemblers for that particular microcontroller. Microchip and Atmel have produced their own compiler, the AVRgcc compiler, which uses the C language for programming. C and its variants C++ and C# are the basis of almost all programs that “work”, as programmers say. The success of Arduino, born in 2005 [38], is due to the simplified programming of the microcontroller on the board. All Arduino boards, that up to today are more than a dozen, are programmed using the Integrated Development Environment (IDE), available for Windows, macOS, and Linux. The programming language is based on a variant of C and C++, so many users are already familiar with it. The IDE interface is very simple and focuses on convenience. Programmers can create functions and subroutines and call them from the main routine, as well as use modules, libraries, and other functions written by thousands of other users.

According to the datasheet mentioned above, the maximum voltage expected from an alkaline battery should be 1.65 V, far from the 5 V maximum of the Arduino converter, so the use of the AREF function is recommended. The sketch for Arduino UNO R3 is available as *Alkaline-Measure-1.1.ino* in the Supplementary Materials file. The sketch is well documented with comments on almost every line and there are two salient parts that need to be described: the first is the use of the AREF function to change the reference voltage to the converter, and the second is the oversampling. All Arduino boards have a pin that produces a stabilized voltage of 3.3 V, used to power shields and modules that work with this voltage; we can use it by connecting it with a wire to AREF to increase the resolution by lowering the reference voltage of the converter from 5 V, which is the voltage normally used, to 3.3 V, which can be obtained by connecting it to the pin indicated above. Arduino UNO R3 has a converter with a resolution of 10 bits, which is 1024 values between 0 V and the Aref value. With the normal value of 5 V we get a resolution of 4.88 mV, while when changing Aref to 3.3 V we get a resolution of 3.22 mV, which is more useful for measuring small variations in the battery. The diagram in Figure 10 shows the presence of a 10 µF/16 V capacitor C1, strongly recommended by the Arduino documentation to stabilize the voltage on AREF.

**Figure 12:**
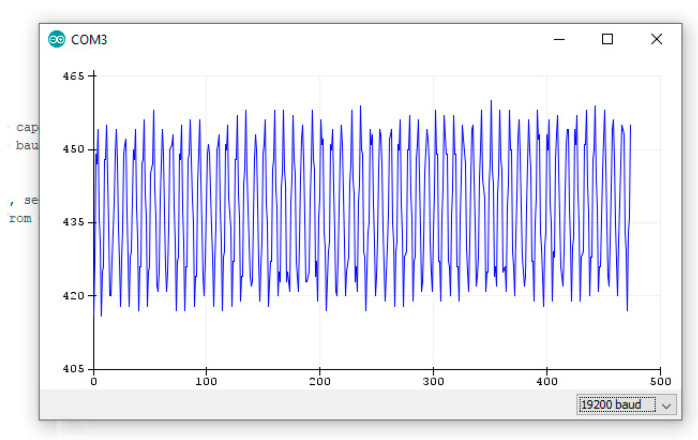
Screenshot of the Arduino IDE serial plotter showing us the noise of the signal read from input pin A0 every 0.25 s for an average of about 0.7 V; on X axis is the count and on Y axis the bits.

The 10-bit resolution of many Arduino boards is often criticized (forgetting that even famous and expensive oscilloscopes, for example, use 10 bits for the Y axis), yet by using oversampling techniques [39,40] it is possible to increase the resolution by 1 bit, and in some cases 2 bits, if the measured signal has random noise, as in our case (see Figure 12).

The proposed sketch makes extensive use of independent routines that can be reused in other contexts following structured programming techniques; among them, one called ReadVolt() oversamples to 11 bits, doubling the resolution, that in our case was already improved by lowering the reference voltage, so by also using the noise we can pass from 3.22 mV to 1.61 mV.

## 3. Results and Discussion

A complete study on the state of charge of exhausted batteries should start with the collection of hundreds, perhaps thousands, of batteries of commercial formats such as LR03/AA, LR06/AA, LR14/C, LR20/D, 6LR61/9V, without forgetting the less common formats such as 3LR12 4.5 V, 4R25X 6 V, 2R10 3 V [41]. Such an approach is very time-consuming, especially considering that for the latter it is difficult to obtain a significant sampling. Therefore, in this paper, the collection was focused only on the LR06/AA format, one of the most widespread, with 706 batteries out of 1021, as reported by Sabbaghi and Behdad [7]. Electronics stores, large supermarkets and shopping centers have special containers for the collection of exhausted batteries, where customers properly dispose of their batteries. In a representative sampling for convenience [42], all AA batteries that were easily accessible in the disposal containers were collected by taking approximately 20 batteries from each.

**Figure 13:**
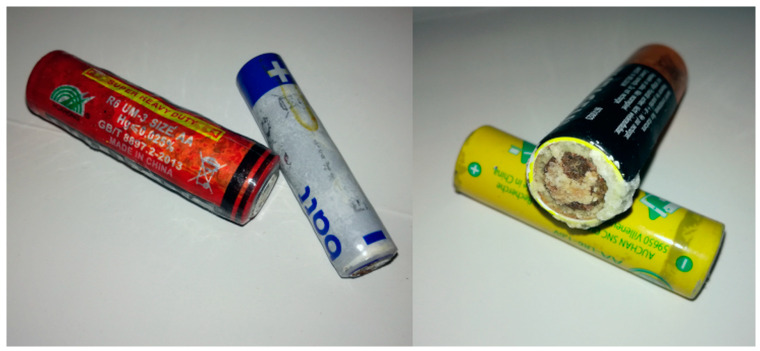
Some alkaline batteries immediately discharged because visibly damaged; four different brands.

During the collection, the clearly or partially damaged batteries (Figure 13) were immediately discarded and only those that appeared to be in perfect appearance and condition were collected; a total of 158 batteries were sampled, some of which even appeared new. Therefore, the actual percentage of damaged batteries among those disposed of in recycle bins cannot be quantified but it can be estimated at around 10%.

The 158 batteries belong to 31 brands, some very widespread and therefore well represented in terms of number of samples, while others are present with only one example; the residual charge under low load of each can be estimated from values reported in the Excel file present in the Supplementary Materials file.

**Figure 14:**
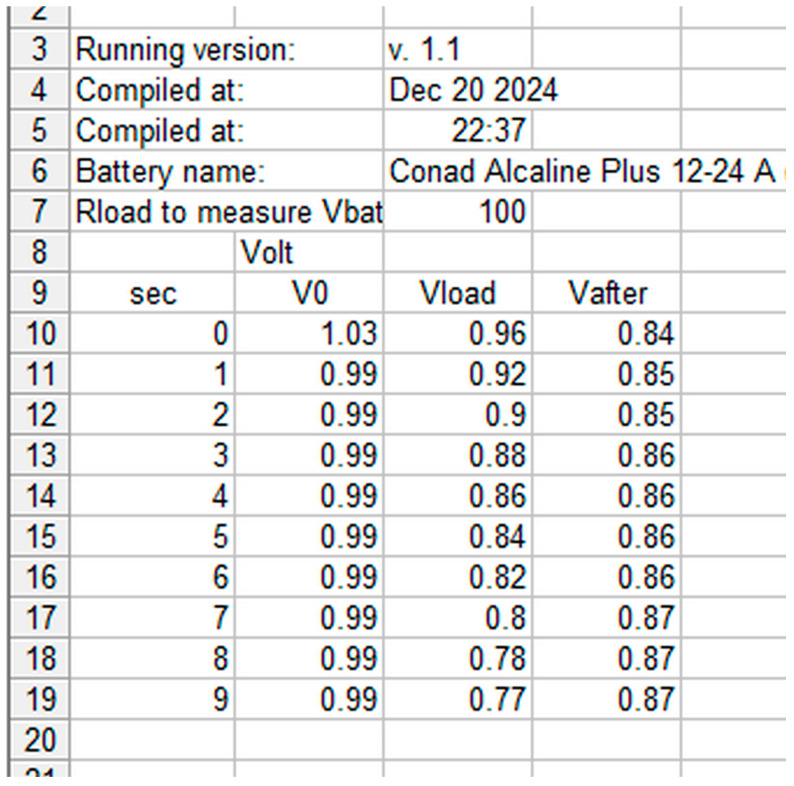
Excel screenshot example showing the table obtained for a battery undergoing measurement.

The measurement protocol is described in Methods and a table like in the screenshot reported in Figure 14 was obtained for each battery by converting the .txt file produced by Arduino into an Excel file.

**Figure 15:**
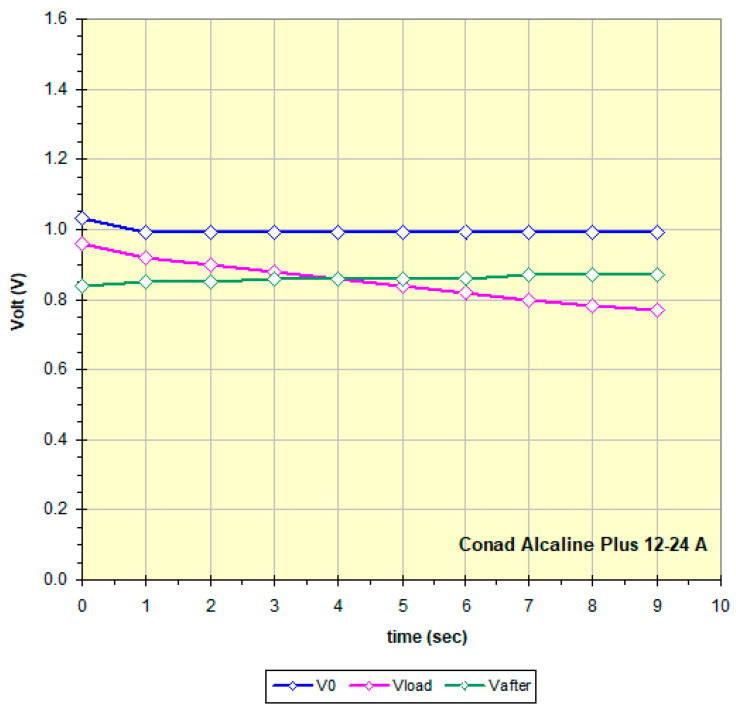
Curves obtained following the measure protocol described in Methods for a battery with a high enough residue charge (data in Figure 14).

Each battery is described in the file with the measurement date, the name that includes the manufacturer, the expiration date (when present), the model and, for samples of the same model, a unique identification code. No rule seems to be applied regarding the date: some manufacturers explicitly indicate the expiration date, others report both the production date and the expiration date with time intervals of 5, 7 and 10 years, and others still only a date that in some cases is assumed to be the production date. Often the dates are well hidden in engravings on the metal of the negative pole. The data from the table are used to draw the discharge graph; see for example Figure 15, obtained from the data in Figure 14.

The curves in Figure 15 show the battery voltage measured every 1 s: blue: at high impedance (remains constant at about 1 V), at the end it disconnects for 5 s; purple: 100 ohm Rload, at the end the battery is disconnected for 10 s; green: again at high impedance.

**Figure 16:**
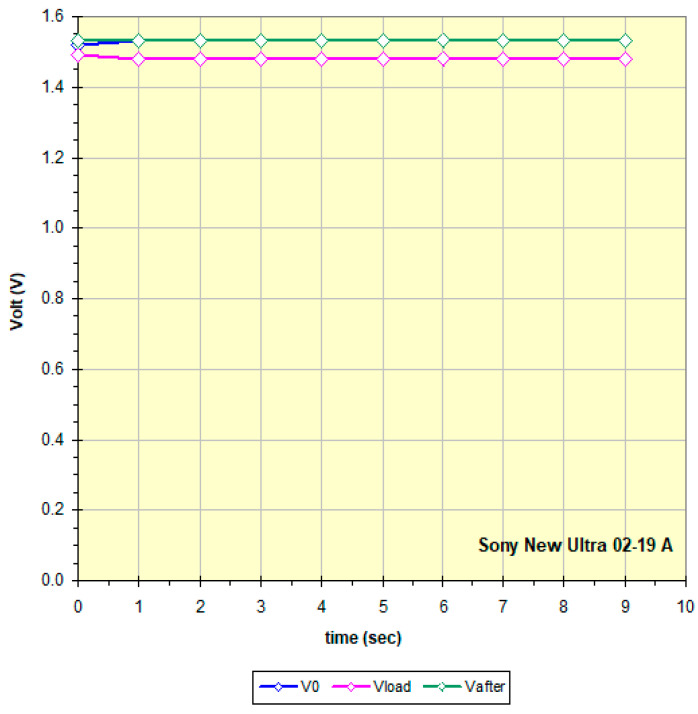
An example of a discarded battery even though it is as good as new.

**Figure 17:**
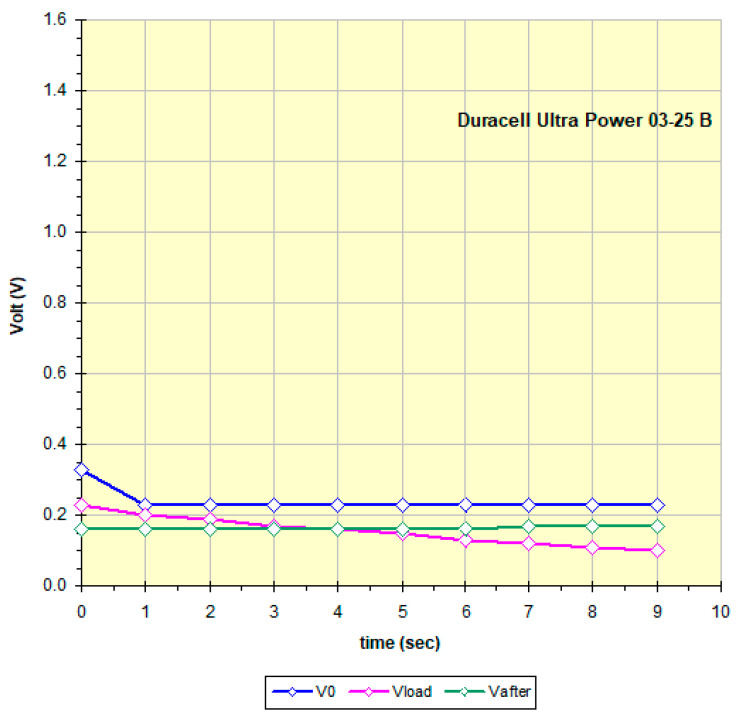
An example of a battery with some residual voltage but probably at the end of its life.

All the graphs obtained by measuring the 158 batteries have a trend similar to that of Figure 14 which is therefore considered “typical”: a constant voltage at no load (high impedance), a slow discharge if current is requested, and after a rest period, the battery recovers and from a new measurement at high impedance the green curve is obtained which probably by increasing the rest time would get closer to the blue curve. The two relays and the 100 ohm Rload were chosen and the times optimized precisely to highlight this behavior. The data in Figure 14 and Figure 15 show that this battery is an excellent candidate for a recharging attempt. To better define the residual charge under low load status of the batteries, the final points of the three curves in Figure 15 were considered and that obtained at 100 ohm Rload was chosen as most significant. Each battery has a different behavior, maintaining a trend as in Figure 15 but showing different slopes and values perhaps linked to the different chemical composition and the different discharge process undergone. For example, Figure 16 refers to a practically new battery, perhaps discarded by mistake after noting the non-functioning of the equipment and thinking it was due to the battery. Figure 17 instead shows a battery practically at the end of its life, therefore not being a good candidate for recharging. Some of the graphs show non-compliant trends whose study is beyond the scope of this work, such as the Maxell China 06-19 A battery (graph in the Excel file in Supplementary Materials); it may be that this trend depends on the fact that the battery, although it still has a fair amount of residual charge under low load, is very old; it is not clear whether the date shown, 2009, is of production and therefore certainly expired or even of expiration.

**Figure 18:**
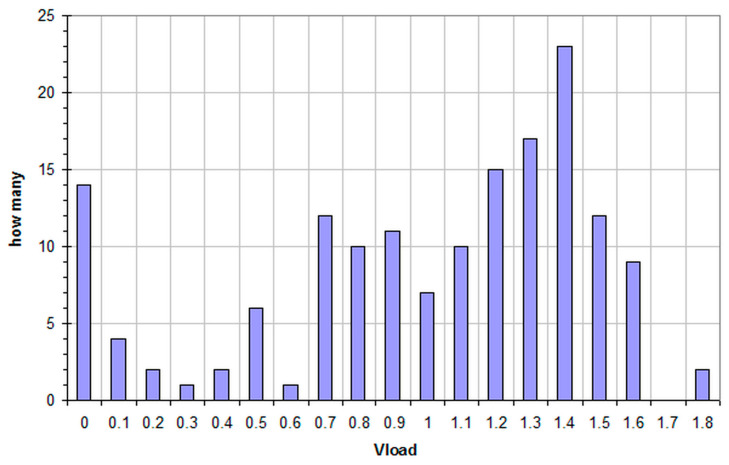
Voltage of all the 158 batteries, 31 brands, AA alkaline, measured at the end of the measure time under load of 100 ohm.

Obviously we do not show the graphs of all 158 measured batteries but fully respecting the open source paradigm, the Excel file present in the Supplementary Materials shows the final values of the three curves obtained for each of them and in Table 1 and Figure 18 some statistical data are reported.

From the table and Figure 18 we see many batteries (about a third) that, although discharged, still have a residual charge under low loads of 1.3, 1.4 and even 1.5 V; moreover, over 5.7% seem to be practically new. It is important to note that these statistics do not include all batteries discharged in the appropriate containers; as already mentioned, those visibly damaged and therefore unusable for our measurements aimed at possible recharging (about 10%) were immediately discharged back into the container. In the attached Excel file we also see packs of two or four batteries that were discharged and then sampled by us keeping them together and in these groups we often notice that only one battery is at the end of its life while the others or all the other three are still usable. A typical case is that of four AA lithium batteries, for two of these the measured voltage is 1.8 V (energizerLithA and LithB) while for the other two we measure 0 V (energizerLithC and LithD). In a world that aims at a circular economy and that has already overcome the 3R paradigm by increasingly focusing on reuse and recycling [43], such a waste of energy [7,8] seems really nonsense. Numerous scientific studies analyze battery recycling in terms of its environmental, technological, economic, and social aspects. These studies examine the recycling of battery components, metal extraction, and energy costs, as well as the cost/benefit ratio [44], but until they are commercially sustainable, reuse first and recycling second is the only viable option.

All the batteries used for the measurements and with a resulting too low charge were discharged according to the Italian regulations for the recycling of exhausted batteries, while the others were kept for the recharging attempt.

## 4. Conclusions

Even though 158 batteries can be considered a small sample, we have obtained good statistics that show 48.7% of batteries with a residual voltage, under low load, between 1.2 and 1.6 V. Taking into account that, according to the literature, the recharge of alkaline batteries appears possible for a residual charge higher than 60% [6], we can affirm that over 67% of the batteries we sampled are good candidates for a recharging attempt (Table 1 and Figure 18). Our handmade apparatus, combined with the protocol we optimized, has proven to be a safe and economical alternative to more expensive and complex techniques for measuring the residual charge of alkaline batteries. The obtained curves, in addition to providing data on the remaining charge, describe the behavior of the batteries and evidence differences between them that can be better studied in future research.

An attempt to safely recharge the aforementioned batteries is instead a must and the purpose of the next already running work. The measurements we here presented will be performed before and after the charging process to calculate its efficiency. Although no risks, such as leaks, occurred during our measurements, these should certainly not be performed by ordinary battery users but only in a research or teaching laboratory. In the latter case, teacher supervision is required both for safety and for detailed explanations of the procedure and the instrument’s schematic. The common battery user should be instead advised to buy a Battery Check, better if a “passive” model that uses the battery under measurement for its operation (Amazon, BT-168 Battery Tester, requiring no battery for operating) so as to identify a flat battery together with if the three others are still usable, perhaps for a mouse, a wall clock or for a radio alarm clock, and so obtain, over the environmental advantages, a quick economic advantage starting from the recovery of the tester cost.

## Figures and Tables

**Table 1:** Statistics of residue charge of 158 AA alkaline battery.

Volt	How Many	%
0	14	8.86
0.1	4	2.53
0.2	2	1.27
0.3	1	0.63
0.4	2	1.27
0.5	6	3.80
0.6	1	0.63
0.7	12	7.59
0.8	10	6.33
0.9	11	6.96
1.0	7	4.43
1.1	10	6.33
1.2	15	9.49
1.3	17	10.76
1.4	23	14.56
1.5	12	7.59
1.6	9	5.70
1.7	0	0.00
1.8 *	2	1.27
count	158	100.00

* Lithium battery; other 2 are included with voltage near to 0 V.

## Data Availability

All measured data and software are available in the Supplementary Materials.

## Supplementary Materials

The following supporting information can be downloaded at https://www.mdpi.com/article/10.3390/mps9020066/s1, All data, Arduino sketch (software) and circuit are available in the Supplementary Materials. The files are: (1) *Alkaline-Measure-1.1.ino*: The sketch and the software written for the Arduino Uno R3, but which runs smoothly on other Arduino AVRs. The sketch was written using Arduino IDE 1.8.9 to ensure broader compatibility, but it runs and compiles smoothly on the latest version of the IDE as of November 2025. (2) *Test-Battery-v1.1_AA-Panasonic-Everyday-01-29-G.txt* is an example file produced by the software/sketch on the Arduino IDE Serial Monitor. (3) *Alkaline-Measure-Extract-v1.1.xls*: An Excel file (Excel 2003 for greater compatibility) that converts the data from the above file (on the first and second sheets) and collects all the measurements from the 158 batteries. (4) *flowchart-by-text.pdf*: An MS word file with text description of flowchart. (5) *Duracell-permission.htm*: Duracell Italia’s response to our request for permission to cite their technical data sheet. (6) *Alkaline-measure-50-100-200.xls* tests conducted at different Rload values to optimize performance. (7) *Alkaline-measure-3.3Vcalibration(3x).xls* description of instrument calibration, also for educational purposes. All files are placed in a single .ZIP file.
