# Supplementary material for: Proposal for a Protocol and a Handmade Arduino-Based and Open Source Device for Measuring the Residual Charge of Alkaline Batteries in View of an Attempt to Recharge Them
Source: Methods Protoc. 2026 Apr 19;9(2):66. doi: 10.3390/mps9020066 (PMC13118361; doi:10.3390/mps9020066)
Supplement: Supplementary file 1 [file mps-09-00066-s001.zip › Duracell-permission.htm]

Gentile Sig. Visco,  
  
Grazie per la sua pazienza.  
  
La vorremmo avvisare che puo' utilizzare il grafico richiesto in maniera generica sotto citazione completa dell'azienza e del nome del documento con fonte.  
  
Speriamo di esserle stati di aiuto, se ha bisogno di altro ci faccia sapere.  
  
Nell’attesa le inviamo cordiali saluti,  
  
Dario  
Servizio Consumatori Duracell  
  
Hai bisogno di contattarci? Per favore clicca qui

> From: CntrProf. G. Visco   
> To: servizi@duracelldirect.it  
> Subject: richiesta cessione Copyright  
> Copies to: richiesta@duracelldirect.it  
> Date sent: Fri, 20 Jun 2025 14:36:19 +1  
>   
> Spett Duracell Italia  
>   
> Vi scriviamo dall´Universita´ La Sapienza, Dipartimento di Chimica, Roma, Italy.  
>   
> Uno dei nostri principali filoni di ricerca e´ l´elettrochimica.  
>   
> Stiamo pubblicando un lavoro dal titolo "Measure of residue charge of primary, alkaline, battery with an Open-Source Arduino project," sulla rivista Metrology dell´editor MDPI.  
>   
> Per il lavoro abbiamo utilizzato la figura nel angolo in alto a destra della seconda pagina del file.  
> https://www.duracell.com/wp-content/uploads/2020/02/MN15US11191.pdf  
>   
> L´editor MDPI ci chiede il permesso del Copyright Owner per questa figura ed eccoci a scrivervi per ottenerlo.  
>   
> Uno dei nostri ultimi lavori in elettrochimica pubblicati e´ il seguente  
> https://www.mdpi.com/2673-4532/4/2/17  
>   
> In allegato sia il datasheet che la figura.  
>   
> Cordiali saluti
