# Supplementary material for: Proposal for a Protocol and a Handmade Arduino-Based and Open Source Device for Measuring the Residual Charge of Alkaline Batteries in View of an Attempt to Recharge Them
Source: Methods Protoc. 2026 Apr 19;9(2):66. doi: 10.3390/mps9020066 (PMC13118361; doi:10.3390/mps9020066)
Supplement: Supplementary file 1 [file mps-09-00066-s001.zip › flow-chart-by-text.pdf]

Paper: Proposal for a protocol and a handmade device Arduino based and Open Source, for measuring the residual charge of alkaline batteries in view of an attempt to recharge them

a) start,

b) press reset on Arduino;

c) make variables setting;

d) send header and values from Arduino to the computer through USB;

e) close the first relay connecting battery to Arduino;

f) measure the battery voltage at impedance >100 Mohm;

g) 1 second delay;

h) measure the battery, repeat points g-h 10 time and send values to PC;

i) 5 seconds delay;

j) close the second relay connecting battery to the 100 ohm load;

k) 1 second delay;

l) measure the battery, repeat points k-l 10 time and send values to PC;

m) open the second relay to leave only the connection battery to high impedance;

n) 10-second delay;

o) 1-second delay;

p) measure the battery, repeat points o-p 10 time and send values to PC;

q) open the first relay to disconnect the battery;

r) end.
